# Posterior Pharyngeal Follicles in a Woman with Breakthrough SARS-CoV-2 Infection

**DOI:** 10.4269/ajtmh.21-1241

**Published:** 2022-03-14

**Authors:** Hidenori Takahashi

**Affiliations:** Department of Infection Control and Prevention, Showa General Hospital, Tokyo, Japan

**Figure 1.**
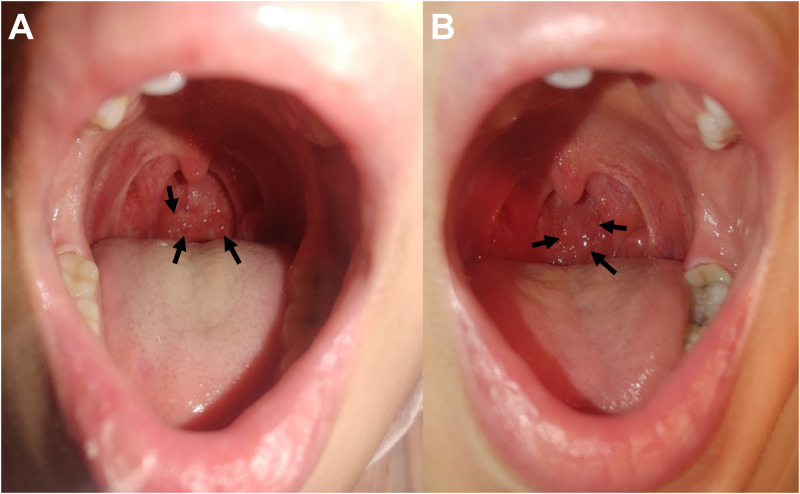
Photographs of the pharyngeal follicles on the posterior wall of the pharynx of a patient with breakthrough severe acute respiratory syndrome coronavirus 2 infection. (**A**) Pharyngeal follicles (arrows) on admission, 1 day after symptom onset. (**B**) Pharyngeal follicles (arrows) at hospital discharge 13 days later. The follicles are enlarged, although the patient was asymptomatic by the time of discharge. This figure appears in color at www.ajtmh.org.

A 24-year-old previously healthy female nurse working in a surgical ward who had received the second dose of BNT162b2 (Pfizer-BioNTech COVID-19 vaccine) 2 months earlier presented with a 1-day history of fever and sore throat. On examination, she had a body temperature of 38.3°C and an oxygen saturation level of 96% breathing room air. Her pharynx was swollen and erythematous, with small red follicles on the posterior wall (Figure 1A). She tested positive for SARS-CoV-2 and negative for 21 other respiratory viruses/bacteria on a BioFire Respiratory 2.1 Panel (bioMérieux, Marcy-l’Ėtoile, France). She was diagnosed with COVID-19 and hospitalized. She developed hoarseness and dysosmia on hospitalization days 3 and 5, respectively. Her symptoms resolved within 9 days without any drug therapy and without respiratory symptoms, hypoxia, or pneumonia. She had to stay in the hospital and undergo a COVID-19 viral antigen rapid test, and returned to work only when repeated negative results were obtained on days 12 and 13 of hospitalization. She was discharged on hospitalization day 13, despite enlarged follicles (Figure 1B). At her 2-month follow-up, the follicles had disappeared.

Pharyngeal lymph follicles are commonly seen in upper respiratory tract infections, such as those caused by the influenza virus, adenovirus, echovirus, and mycoplasma (all detectable with the BioFire Respiratory 2.1 Panel), and they are useful for early diagnosis.^1^^–^^3^ Similarly, they are also seen in SARS-CoV-2 infection^4^; however, they might not receive much attention from clinicians. The pharynx plays important roles in viral entry, replication, and transmission in SARS-CoV-2 infection, and follicle growth/atrophy reflects viral proliferation/shedding.^1^^,^^5^ The relationships between pharyngeal lymph follicles and COVID-19 vaccine are not well established, but vaccinated patients are known to shed infectious SARS-CoV-2.^6^

During the COVID-19 pandemic, clinicians might have opportunities to examine the oral cavity. Clinicians usually examine the oral cavity in patients with fever and upper respiratory symptoms such as the common cold or influenza, which display symptoms similar to those of a SARS-CoV-2 breakthrough infection.^1^^,^^7^ Furthermore, some hospitals continue to conduct oropharyngeal swab polymerase chain reaction testing, which enables oral cavity examination in tandem.^4^

Clinicians should pay attention to pharyngeal follicles, even in patients who have been fully vaccinated, as it could help detect SARS-CoV-2 and prevent nosocomial infections, at least as depicted by this case.
